# Application of next-generation sequencing for detecting *Mycoplasma* contamination in veterinary vaccines

**DOI:** 10.3389/fvets.2025.1657098

**Published:** 2025-10-10

**Authors:** Su-Min Go, Yeon-Kyeong Lee, Jin-Ju Nah, Hyun-Ok Gu, Il Jang, Min-Goo Seo

**Affiliations:** ^1^Veterinary Drugs and Biologics Division, Animal and Plant Quarantine Agency, Gimcheon-si, Gyeongsangbuk-do, Republic of Korea; ^2^College of Veterinary Medicine and Institute for Veterinary Biomedical Science, Kyungpook National University, Daegu, Republic of Korea; ^3^Bacterial Disease Division, Animal and Plant Quarantine Agency, Gimcheon-si, Gyeongsangbuk-do, Republic of Korea

**Keywords:** *Mycoplasma* detection, next-generation sequencing, reference mapping, veterinary vaccines, vaccine quality control, PCR limitations, metabarcoding

## Abstract

Ensuring the safety and efficacy of veterinary vaccines requires reliable methods for detecting microbial contamination, particularly from *Mycoplasma* species, which pose a significant risk in cell-culture-derived vaccines. In the Republic of Korea, polymerase chain reaction (PCR) is predominantly used for *Mycoplasma* testing due to its faster turnaround compared to culture-based methods. However, in combination with vaccines containing *Erysipelothrix rhusiopathiae* and classical swine fever virus, PCR is rendered ineffective because of cross-reactivity between *Mycoplasma* universal primers and *E. rhusiopathiae*, resulting in non-specific amplification. This limitation necessitates reliance on the labor-intensive culture method, underscoring the need for more accurate and efficient alternatives. This study aimed to develop and validate next-generation sequencing (NGS)-based methods for detecting *Mycoplasma* contamination in veterinary vaccines and to compare their performance with that of PCR. Five species, including *Acholeplasma laidlawii* (genus *Acholeplasma*) and four *Mycoplasma* species—*Mycoplasma fermentans*, *Mycoplasma orale*, *Mycoplasma hyorhinis*, and *Mycoplasma synoviae*–were spiked into samples containing *E. rhusiopathiae*, a common vaccine component. Two NGS-based approaches were evaluated: (1) a reference-mapping method incorporating two-step alignment and *de novo* assembly, and (2) a 16S rRNA-based metabarcoding analysis using DADA2 and Qiime2. The reference-mapping method effectively filtered non-specific reads and accurately reconstructed *Mycoplasma*-derived contigs, whereas the metabarcoding approach enabled taxonomic profiling with quantitative resolution. The detection limits of NGS-based methods were substantially lower than those of PCR, demonstrating improvements of up to 100-fold depending on the species. Notably, omission of the initial mapping step resulted in excessive non-specific contig formation, highlighting the importance of the dual-step reference-mapping strategy. Although metabarcoding provided valuable abundance data, it was more prone to non-specific hits due to limited read overlap. In conclusion, the reference-mapping method demonstrated superior sensitivity, specificity, and quantification compared to both conventional PCR and metabarcoding, supporting its use as a robust tool for vaccine quality control. Implementing NGS-based detection methods could significantly enhance the safety and effectiveness of veterinary vaccines, ultimately enhancing vaccine quality control.

## 1 Introduction

Before veterinary vaccines can be marketed, they must undergo testing to ensure compliance with quality standards, safety regulations, and efficacy requirements established by regulatory authorities (1). These tests are conducted both in the countries where the products are approved for sale and internally by manufacturers. Commercial vaccines are vulnerable to contamination by various adventitious agents during the manufacturing process, including bacteria, viruses, fungi, and *Mycoplasma* species. Among these contaminants, *Mycoplasma* has emerged as a particularly concerning issue due to its potential impact on animal health and vaccine effectiveness (2).

*Mycoplasma* species are a type of bacteria lacking cell walls and are capable of infecting a range of animal hosts, resulting in substantial economic losses in the agricultural sector (3). Their lack of a cell wall makes them inherently resistant to antibiotics that target cell wall synthesis, complicating treatment efforts (4). *Mycoplasma* can also contaminate cell cultures and biological products without causing visible signs of infection, making detection difficult. Conventional methods for identifying *Mycoplasma* contamination include culture-based techniques and polymerase chain reaction (PCR) assays, both of which are used by the Animal and Plant Quarantine Agency (APQA) in the Republic of Korea for testing veterinary biologicals. Culture-based methods are widely recognized for their ability to detect viable organisms, but they are time-consuming, often requiring up to 28 days for results (1, 5). This delay presents challenges for timely quality control during vaccine production. At APQA, a broad-range PCR assay targeting multiple *Mycoplasma* species is employed as the initial screening method. PCR-positive samples are subsequently subjected to culture, and the results of culture are used to make the final determination of *Mycoplasma* contamination.

**Figure 1:**
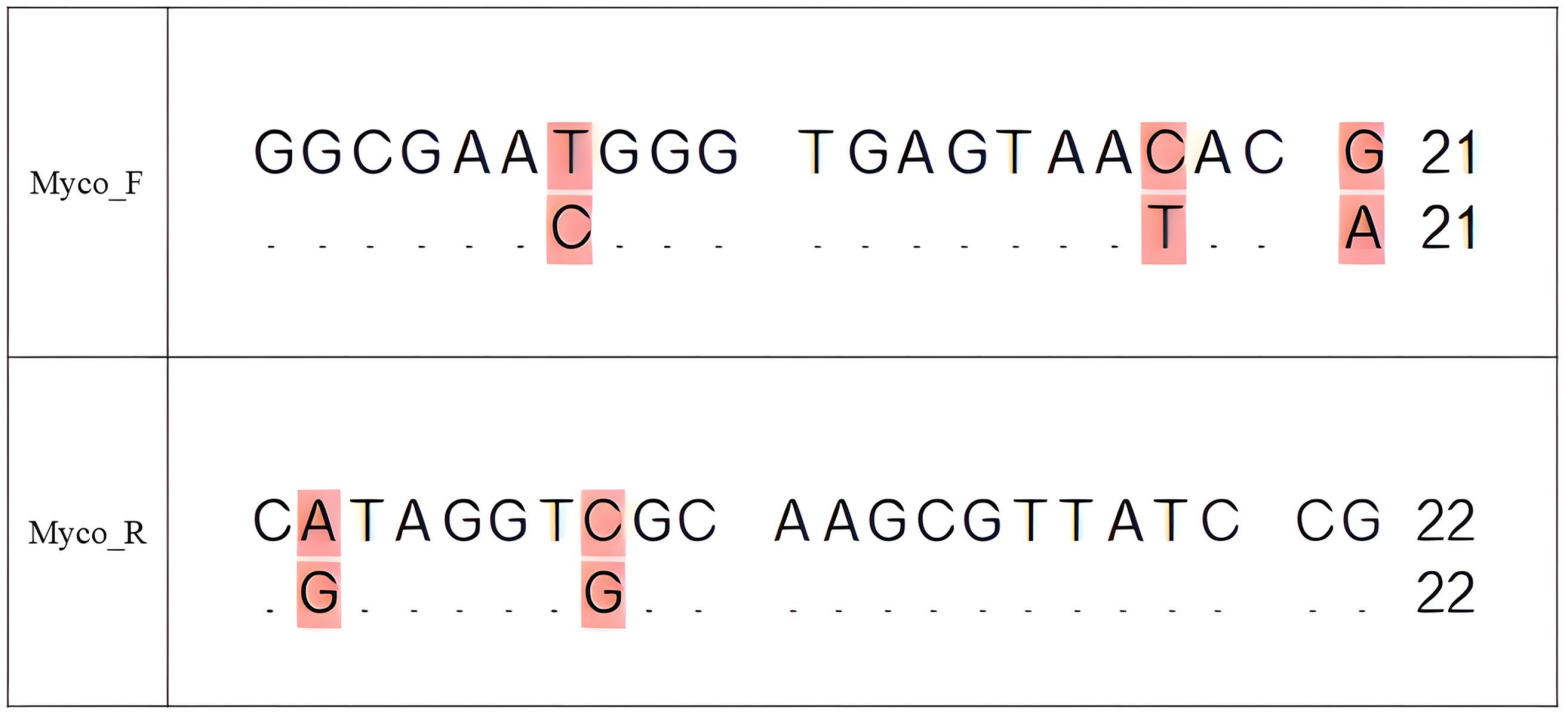
Sequence alignment of the universal Mycoplasma forward (Myco_F) and reverse (Myco_R) primers with the 16S rRNA sequence of *E. rhusiopathiae* (GenBank accession no. NR_040837). For each primer, the upper line represents the primer sequence and the lower line represents the aligned *E. rhusiopathiae* sequence. Identical nucleotides are indicated by dots (“.”) in the lower sequence, while mismatches are shown as their respective bases and highlighted in pink to indicate regions of divergence that may result in non-specific binding. The forward primer aligns to positions 95–115 bp, and the reverse primer aligns to positions 534–555 bp of the *E. rhusiopathiae* 16S rRNA gene. This alignment highlights the potential for cross-reactivity that could generate false positives in PCR assays targeting *Mycoplasma* spp.

During routine national lot release testing of combination vaccines containing *Erysipelothrix rhusiopathiae* (*E. rhusiopathiae*) and classical swine fever virus (CSFV) as antigens, non-specific bands in *Mycoplasma* PCR assays were consistently observed. Analysis of cumulative results from the national lot release tests conducted by APQA in Korea further confirmed that these non-specific findings occurred repeatedly. Basic Local Alignment Search Tool (BLAST) analysis identified these bands as sequences derived from *E. rhusiopathiae*. Alignment of *E. rhusiopathiae* sequences with *Mycoplasma* universal primers revealed near-complete complementarity, with only a few nucleotide mismatches (Figure 1). This finding suggests that genetic similarities between *E. rhusiopathiae* and *Mycoplasma* species can interfere with PCR specificity, resulting in non-specific amplification and potentially compromising the accuracy of *Mycoplasma* testing (6, 7).

Next-generation sequencing (NGS) technologies have recently proven to be highly effective for detecting and identifying microorganisms. NGS offers high sensitivity and the ability to identify a broad range of microbes without requiring prior assumptions about their presence (8). It provides results within hours to days, addressing the significant delays associated with culture-based methods, which rely on microbial isolation and can take several days or weeks. In addition, culture methods often struggle to accurately identify organisms that are slow-growing or fastidious. NGS overcomes these challenges by enabling direct, unbiased sequencing of microbial DNA, thereby delivering faster and more accurate results (9).

This study aims to address the limitations of PCR by developing a more precise and reliable NGS-based approach for detecting *Mycoplasma* contamination in veterinary vaccines. The goal is to resolve issues of non-specific amplification and enable clear differentiation between *Mycoplasma* and *E. rhusiopathiae* in mixed samples. By achieving accurate detection even in the presence of both pathogens, this method is expected to improve the reliability of vaccine quality control.

## 2 Materials and methods

### 2.1 Bacterial strains

The test materials were selected in accordance with the International Cooperation on Harmonisation of Technical Requirements for Registration of Veterinary Medicinal Products guideline 34, which serves as a standard for assessing the safety, quality, and efficacy of veterinary drugs, particularly biological products (Table 1). To assess potential contamination, the guideline mandates the use of five specific organisms belonging to the class Mollicutes. Although these organisms belong to different families, they include one species of *Acholeplasma* (*A. laidlawii*) and four species of *Mycoplasma* (*M. fermentans*, *M. orale*, *M. hyorhinis*, and *M. synoviae*). These species were chosen based on several factors, including antibiotic susceptibility, culture requirements, potential for contamination, and pathogenicity (10). These organisms originate from a range of hosts, including mammals, birds, and humans. The five species used in this study were obtained from the American Type Culture Collection. In addition, *E. rhusiopathiae* was selected as a test material for the development of bioinformatics-based analytical methods due to its genetic similarity to *Mycoplasma*, which makes it difficult to distinguish using PCR. A commercial vaccine approved in the Republic of Korea containing CSFV and *E. rhusiopathiae* antigens was used in the study.

**Table 1:** Bacterial and *Mycoplasma* species used in this study.

Species	Strain
*Acholeplasma laidlawii*	ATCC^a^ 23206
*Mycoplasma fermentans*	ATCC 19989
*Mycoplasma orale*	ATCC 23714
*Mycoplasma hyorhinis*	ATCC 17981
*Mycoplasma synoviae*	ATCC 25204
*Erysipelothrix rhusiopathiae*	NL-11^b^

a ATCC, American type culture collection.

b NL-11, live vaccine strain.

### 2.2 Sample preparation

Initially, both *E. rhusiopathiae* and *Mycoplasma* species were diluted using phosphate-buffered saline. *E. rhusiopathiae* was adjusted to achieve a concentration equivalent to two vaccine doses. Equal volumes (1 mL each) of the *E. rhusiopathiae* vaccine and serial dilutions of *Mycoplasma* species were then mixed. This ensured that the final concentration of *E. rhusiopathiae* in the mixed sample corresponded to a single vaccine dose, while *Mycoplasma* concentrations varied. The spike assay confirmed that *E. rhusiopathiae* remained at 1.6 × 10^9^ CFU/mL, while the starting concentrations of the five *Mycoplasma* species before dilution were as follows: 1.35 × 10^6^ CFU/mL for *A. laidlawii*, 1.05 × 10^6^ CFU/mL for *M. fermentans*, 7.15 × 10^5^ CFU/mL for *M. orale*, 7.95 × 10^3^ CFU/mL for *M. hyorhinis*, and 2.5 × 10^6^ CFU/mL for *M. synoviae*. In addition to the spiked samples, a separate group containing only Mycoplasma, without *E. rhusiopathiae*, was prepared to assess the detection threshold by PCR. All experiments, including sample preparation and downstream analysis, were performed in triplicate to ensure reliability and reproducibility.

Along with the spiked samples, 31 negative vaccine samples—corresponding to half of the 62 vials assigned for quality control at APQA in 2024—were also analyzed. These samples consisted of commercial combination vaccines containing only CSFV and *E. rhusiopathiae*, and were presumed to be free of *Mycoplasma* contamination. The negative field samples were used to verify assay specificity and to serve as negative controls for downstream PCR and NGS-based analyses.

DNA extraction was performed using an automated nucleic acid platform (Maelstrom 4810, TANBead, Taiwan, China) and a magnetic bead-based protocol with the TANBead Nucleic Acid Extraction Kit (TANBead). Following the manufacturer’s instructions, 300 μL of sample and 10 μL of Proteinase K were used as input. The DNA was eluted in 80 μL of elution buffer.

### 2.3 PCR and amplicon sequencing

The 16S rRNA molecule, approximately 1,500 base pairs in length, is a widely conserved sequence that contains essential structural regions along with variable regions that enable differentiation between bacterial species. This makes it a commonly used tool in microbiology for bacterial identification and phylogenetic analysis (7). In this study, primers were designed to amplify the 16S rRNA region (11) with the following sequences (Table 2): universal forward primer (5′-GGC GAA TGG GTG AGT AAC ACG-3′) and universal reverse primer (5′-CGG ATA ACG CTT GCG ACC TAT C-3′). These primers follow the National Regulatory Standards for Veterinary Biologicals established by the Republic of Korea (1). PCR was carried out using the Maxime™ PCR premix (i-Taq, iNtRON Biotechnology) in a total reaction volume of 20 μL comprising 1 μL (10 pmol) of forward primer, 1 μL (10 pmol) of reverse primer, 2 μL of DNA, and 16 μL of distilled water. Thermal cycling conditions included an initial denaturation at 94 °C for 5 min; 30 cycles of denaturation (1 min at 94 °C), annealing (1 min at 60 °C), and extension (1 min 30 s at 72 °C); followed by a final extension at 72 °C for 7 min. PCR products were analyzed by electrophoresis on a 1.5% agarose gel, and the presence of a specific band at 464 base pairs (bp) was confirmed using a UV transilluminator.

**Table 2:** Primer details, including nucleotide sequences, locations, amplicon size, and references.

Primers	Nucleotide sequences (5′ → 3′)	Location	Amplicon size (bp)	Reference
Universal forward	GGCGAATGGGTGAGTAACACG	90–110^a^	464	Wong-Lee and Lovett (11)
Universal reverse	CGGATAACGCTTGCGACCTATG	553–532^a^		

a Numbered according to GenBank accession no. M24579.

The PCR products were submitted to a commercial provider (BIONICS, Daejeon, Republic of Korea) for library preparation, quality control, and sequencing using the BITseq next-generation sequencing service. Sequencing was performed with a target output of 30,000 reads, and data were delivered in FASTQ format containing 150 bp paired-end reads. These reads were used as raw data for downstream analysis.

### 2.4 Reference-mapping analysis

**Figure 2:**
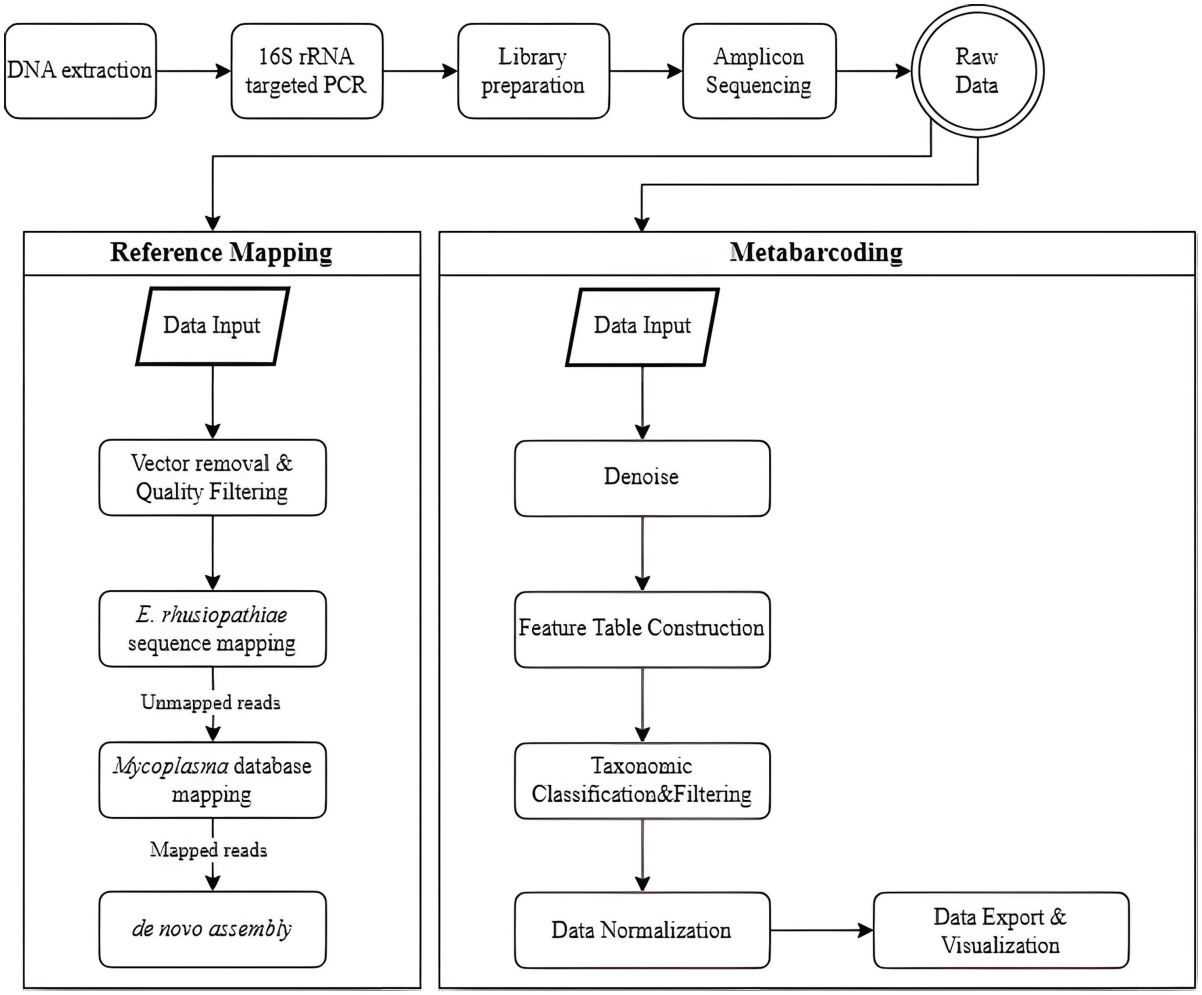
Workflow for preprocessing and analyzing next-generation sequencing (NGS) data, comparing two methods: reference mapping and metabarcoding. In the reference-mapping method, the BBMap and SPAdes tools are used. Raw reads undergo vector removal and quality filtering, followed by sequential mapping-first to the *E. rhusiopathiae* reference genome, then to a *Mycoplasma* spp. database-with mapped reads subjected to *de novo* assembly. In the metabarcoding method, Qiime2 and MicrobiomeAnalyst are employed. Denoising is performed to correct sequencing errors and identify unique amplicon sequence variants (ASVs), which are classified taxonomically. Contaminants, low-confidence ASVs, and unclassified sequences are removed. Normalization and rarefaction are applied to control for sequencing depth before relative abundance is analyzed.

The raw sequencing data were analyzed using two distinct approaches: a reference-mapping pipeline and a metabarcoding workflow, each employing specialized software tailored to its respective analytical purpose (Figure 2). The reference-mapping method was developed to isolate specific strains from heterogeneous samples. BBMap (v39.01) was used to align sequencing reads to predefined reference genomes (12), while SPAdes (v3.13.1) served as the assembler for genome reconstruction (13, 14). Key features of this pipeline included high-accuracy alignment to reference genomes and targeted extraction of *Mycoplasma*-derived contigs.

Prior to analysis, reference sequences for *E. rhusiopathiae* and *Mycoplasma* spp. were compiled. For *E. rhusiopathiae*, the 16S rRNA partial sequence of strain ATCC 19414 was obtained from the NCBI reference sequence database. Concurrently, 16S rRNA gene reference sequences were obtained from the SILVA ribosomal RNA gene database (released ver. 138.2, SSURef NR99) (15). To construct a targeted database for mapping, sequences were filtered to retain only those assigned to the families *Mycoplasmataceae*, *Acholeplasmataceae*, *Metamycoplasmataceae*, and *Mycoplasmoidaceae* according to SILVA taxonomy (Supplementary Data Sheet 1). In parallel, a UniVec database containing adapter, linker, and primer sequences commonly introduced during cloning workflows was incorporated (16). Although the library preparation process did not explicitly confirm the presence of such sequences, vector screening was included as a precautionary measure to mitigate potential contamination and safeguard data integrity. Vector sequence removal and quality filtering are critical in reference mapping to prevent non-specific alignment of contaminant reads to the reference genome. This pre-processing step is essential for reducing false-positive alignments in downstream mapping.

The reference-mapping protocol consisted of four sequential stages, beginning with vector removal and quality filtering. The bbduk.sh script was used for both tasks. In the first stage, reads were aligned to the UniVec database and separated into vector-contaminated reads (outm1, outm2) and cleaned reads (outu1, outu2). The key parameters included *k* = 31 (*k*-mer length of 31) and hdist = 1 (maximum permitted Hamming distance of 1). In the second stage, adapter trimming and quality filtering were performed using the same script with the following parameters: ktrim = r (trimming from the right), *k* = 23 (*k*-mer length of 23), mink = 11 (minimum *k*-mer length of 11), hdist = 1 (maximum permitted Hamming distance of 1), qtrim = rl (quality trimming from both ends), trimq = 20 (quality score threshold of 20), and minlen = 50 (minimum read length of 50 bases).

The next two steps involved reference-based alignment using the bbmap.sh script. In step three, reads that did not align with the *E. rhusiopathiae* reference genome were retained using minid = 0.95 (minimum identity of 95%) and maxindel = 3 (maximum allowable insertion/deletion length of 3 bases). In step four, the remaining reads were aligned to the *Mycoplasma* reference database with stricter parameters: minid = 0.99 (minimum identity of 99%) and maxindel = 3. Genome assembly was then performed using the SPAdes assembler (spades.py) (14). The detection limit of *Mycoplasma* was established by evaluating colony-forming unit (CFU) counts and identifying contig formation at corresponding dilution levels.

To enable accurate comparisons across samples, sequencing depth was normalized using the Total Count (TC) method. This involved calculating the total coverage for each contig (contig length multiplied by average coverage) and dividing it by the total number of reads in the sample (17). This normalization approach minimizes technical bias introduced by varying sequencing depths and enables consistent interpretation across datasets. The formula used is as follows:


$$\begin{array}{l} \text{Normalized Coverage}\\ =\frac{\text{Total Coverage}\;(\text{Contig Length}\times \text{Average Coverage})}{\text{Total Reads in Sample}} \end{array}$$

### 2.5 Metabarcoding analysis

The metabarcoding analysis was conducted using Qiime2 (v2024.05) to examine sequencing data from diverse microbial communities present in the samples (18). The analysis included quality filtering, sequence clustering, and taxonomic classification at the order level to identify and categorize different species. MicrobiomeAnalyst was also used for downstream data visualization and interpretation (19).

Initial sequencing data were processed in Qiime2 (v2024.05) through a series of bioinformatic procedures (15, 18). Raw sequence reads were imported into the pipeline, and quality control steps were applied. This included preprocessing with DADA2 to remove noise, correct sequencing errors, and identify amplicon sequence variants (ASVs), providing a high-resolution representation of microbial diversity. Following denoising, a feature table was constructed to record the occurrence of each ASV across all samples. Taxonomic classification of ASVs was performed by aligning sequences with the SILVA 16S rRNA reference database (15), followed by filtering to remove low-confidence assignments and potential contaminants. The data were then normalized to account for variations in sequencing depth, using methods such as rarefaction to allow comparability between samples (18, 19). Finally, the processed data were exported for statistical analysis and visualized using various tools within MicrobiomeAnalyst (19). We assessed the prevalence of *Mycoplasma* in each sample based on visual outputs and evaluated detection limits accordingly.

## 3 Results

### 3.1 Detection limit of conventional PCR: a benchmark for detection

The detection limits for five *Mycoplasma* species—*A. laidlawii*, *M. fermentans*, *M. orale*, *M. hyorhinis*, and *M. synoviae*—were evaluated (Table 3). Amplification for *A. laidlawii* was consistently observed at 3.13. log CFU/mL across all replicates, with no detection at lower concentrations. *M. fermentans* showed a consistent detection threshold of 3.02 log CFU/mL in all three replicates. Similarly, *M. orale* produced amplification at 3.85 log CFU/mL across all replicates. In the case of *M. hyorhinis*, the detection limit varied slightly, with amplification observed at 2.9, 1.9, and 1.9 log CFU/mL, respectively; however, 1.9 log CFU/mL was the most consistent threshold. *M. synoviae* demonstrated reproducible amplification at 3.4 log CFU/mL across all replicates. Collectively, these results indicate variation in PCR detection efficiency among *Mycoplasma* species, with amplification generally decreasing as target concentrations decline.

**Table 3:** Detection limits of Mycoplasma species using three analytical methods: conventional PCR, reference mapping, and metabarcoding.

Species	Detection limit (log CFU/mL)		
	PCR	Reference-mapping	Metabarcoding
*A. laidlawii*	3.1 ± 0^a^	1.6 ± 0.6	1.6 ± 0.6
*M. fermentans*	3.0 ± 0	1.0 ± 0	0.7 ± 0.6
*M. orale*	3.9 ± 0	2.5 ± 0.9	1.2 ± 0.9
*M. hyorhinis*	2.2 ± 0.5	0.2 ± 0.5	0.2 ± 0.5
*M. synoviae*	3.4 ± 0	0.7 ± 0.5	−0.3 ± 0.5

a Values are presented as mean ± standard deviation.

### 3.2 Reference mapping analysis for high-precision detection

The reference-mapping method was applied to assess its performance in detecting multiple *Mycoplasma* spp. in spiked samples. The results present an overview of the read retention at each step of the reference-mapping process and demonstrate how mapped read counts vary according to bacterial concentration.

Sequencing yielded consistently high read counts across all species: 27,633 reads for *A. laidlawii*, 33,496 for *M. fermentans*, 33,210 for *M. orale*, 36,347 for *M. hyorhinis*, and 33,904 for *M. synoviae*, with 100% read retention at the initial stage. After vector sequence removal and quality trimming, nearly all reads were retained, indicating minimal data loss and high initial sequence quality.

Following the first step against *E. rhusiopathiae,* read counts decreased significantly across species: *A. laidlawii* retained 2.6% (726 reads), *M. fermentans* 3.4% (1,129 reads), *M. orale* 4.2% (1,410 reads), *M. hyorhinis* 13.0% (4,729 reads), and *M. synoviae* 7.1% (2,419 reads). A second mapping step against the *Mycoplasma* database further reduced reads, yielding 0.3% (84 reads) for *A. laidlawii*, 1.3% (453) for *M. fermentans*, 1.7% (577) for *M. orale*, 8.3% (3,018) for *M. hyorhinis*, and 2.5% (863) for *M. synoviae* (Table 4). These findings demonstrate the effectiveness of the two-step reference-mapping strategy in removing non-specific reads and emphasize the value of sequential mapping for accurate downstream analysis.

**Table 4:** Summary of sequencing data for five *Mycoplasma* spp. at *α* × 10^1^ CFU/mL.

Steps	Description	Output reads (percentage to raw reads)				
		*A. laidlawii*	*M. fermentans*	*M. orale*	*M. hyorhinis*	*M. synoviae*
1	Raw reads	27,633 (100%)	33,496 (100%)	33,210 (100%)	36,347 (100%)	33,904 (100%)
2	Vector removal and Quality trimming	27,627 (99.9%)	33,459 (99.8%)	33,207 (99.9%)	36,340 (99.9%)	32,944 (97.1%)
3	Reference mapping^a^ (*E. rhusiopathiae*)	726 (2.6%)	1,129 (3.3%)	1,410 (4.2%)	4,729 (13.0%)	2,419 (7.1%)
4	Reference mapping (*Mycoplasma* DB)	84 (0.3%)	453 (1.3%)	577 (1.7%)	3,018 (8.3%)	863 (2.5%)

The table shows read counts after each processing step and the percentage retained relative to raw reads. Specific α values are as follows: *A. laidlawii* = 1.35, *M. fermentans* = 1.05, *M. orale* = 7.08, *M. hyorhinis* = 7.94, *M. synoviae* = 2.51.

a Output represents unmapped reads after mapping to the *E. rhusiopathiae* reference genome.

### 3.3 Metabarcoding analysis for comprehensive detection

The metabarcoding method was employed to evaluate the relative abundance of *Mycoplasma* spp. across various dilution levels, offering insights into sample composition and detection limits, For comparison, a table presenting both relative coverage and relative abundance is included (Table 5).

**Table 5:** Relative coverage and relative abundance of five *Mycoplasma* species across dilution levels (CFU/mL).

CFU/mL	*A. laidlawii*		*M. fermentans*		*M. orale*		*M. hyorhinis*		*M. synoviae*	
	Relative coverage^a^	Relative abundance^b^	Relative coverage	Relative abundance	Relative coverage	Relative abundance	Relative coverage	Relative abundance	Relative coverage	Relative abundance
α × 10^5^	$\frac{7,123\pm 494}{29,978\pm 2,567}$	88.0	$\frac{8,579\pm 1,022}{32,697\pm 2,698}$	94.4	$\frac{8,356\pm 151}{32,718\pm 1,207}$	82.2	NT^c^	NT	$\frac{9,726\pm 855}{34,090\pm 1,537}$	92.7
α × 10^4^	$\frac{5,017\pm 672}{29,268\pm 1,647}$	57.1	$\frac{6,462\pm 568}{30,168\pm 1,996}$	77.1	$\frac{4,492\pm 234}{34,171\pm 1,337}$	78.4	NT	NT	$\frac{7,784\pm 2,421}{33,059\pm 3,371}$	77.1
α × 10^3^	$\frac{1,360\pm 182}{28,150\pm 3,950}$	16.5	$\frac{3,108\pm 699}{32,259\pm 792}$	35.4	$\frac{583\pm 522}{33,716\pm 1,252}$	41.1	$\frac{8,783\pm 795}{31,972\pm 488}$	89.3	$\frac{4,452\pm 1,214}{35,688\pm 348}$	42.1
α × 10^2^	$\frac{450\pm 325}{31,034\pm 3,453}$	3.8	$\frac{831\pm 285}{32,118\pm 1,690}$	9.1	$\frac{317\pm 441}{32,182\pm 1,987}$	7.4	$\frac{8,151\pm 899}{34,957\pm 861}$	74.0	$\frac{1,286\pm 276}{35,049\pm 1,014}$	12.6
α × 10^1^	$\frac{31\pm 34}{27,633\pm 789}$	0.3	$\frac{146\pm 12}{33,496\pm 1,931}$	1.3	$\frac{0\pm 0}{33,210\pm 1,107}$	4.9	$\frac{4,460\pm 277}{33,770\pm 1,699}$	40.3	$\frac{262\pm 27}{33,904\pm 1,342}$	2.3
α × 10^0^	$\frac{0\pm 0}{32,006\pm 2,469}$	0	$\frac{0\pm 0}{32,394\pm 2,301}$	0	$\frac{0\pm 0}{33,350\pm 1,658}$	1.8	$\frac{1,057\pm 498}{36,347\pm 85}$	8.8	$\frac{72\pm 110}{31,929\pm 1,917}$	0.7
α × 10^−1^	$\frac{0\pm 0}{32,464\pm 1,755}$	0	$\frac{0\pm 0}{32,062\pm 1,923}$	0	$\frac{0\pm 0}{32,302\pm 1,051}$	0.5	$\frac{91\pm 81}{34,128\pm 2,025}$	0.6	$\frac{0\pm 0}{30,836\pm 6,950}$	0

Values represent the mean across replicates. Dilution levels (e.g., *α* × 10^5^) reflect serial dilutions based on each species’ initial concentration: *A. laidlawii* = 1.35, *M. fermentans* = 1.05, *M. orale* = 7.08, *M. hyorhinis* = 7.94, *M. synoviae* = 2.51 (log CFU/mL).

a Relative coverage calculated as average coverage divided by total reads.

b Relative abundance calculated as the proportion of species within the sample.

c NT, not tested.

Relative coverage, derived from the reference-mapping method, refers to the proportion of reads mapped to a reference genome out of the total reads in a sample. It is calculated as the sum of the average coverage values across all genome regions divided by the total read count. Relative abundance, in contrast, reflects the proportion of each species within the total microbial population, providing information on species diversity and distribution. Although the relative coverage data are presented for reference, the primary focus is on abundance patterns obtained from the metabarcoding method.

For *A. laidlawii*, the relative abundance at the highest concentration (*α* × 10^5^ CFU/mL) was 88.0%. As the dilution increased, abundance dropped sharply, reaching 0% at *α* × 10^0^ and *α* × 10^−1^ CFU/mL, where no contigs were detected. *M. fermentans* showed a similar pattern, with 94.4% abundance at α × 10^5^ CFU/mL, decreasing to 0% at the lowest concentrations. *M. orale* followed this trend, starting at 82.2% and declining to 0% with increasing dilution. *M. synoviae* exhibited the highest starting abundance (92.7%) at α × 10^5^ CFU/mL, which also dropped to 0% at lower levels. In contrast, *M. hyorhinis*, which began testing at α × 10^3^ CFU/mL due to limited starting material, showed 89.3% abundance at that level. Higher concentration experiments were not conducted for this species, resulting in “Not Tested” values. Despite this, the metabarcoding successfully detected *M. hyorhinis* at lower concentrations, demonstrating its utility in identifying less abundant species in spiked samples. Non-specific reads from genera such as *Bacillus* and *Staphylococcus* were also detected.

The detection limits of *Mycoplasma* spp. were compared across three methods: conventional PCR, the reference-mapping method, and the metabarcoding method (Table 3). Across all three methods, standard deviation remained below one log unit across triplicate experiments. PCR exhibited the highest detection thresholds, while both NGS-based methods yielded lower detection limits across all species.

### 3.4 Analysis of negative vaccine samples for Mycoplasma contamination

Initial reference-mapping method yielded *mycoplasma*-specific contigs in 5 of the 31 negative vaccine samples. The contigs were 447, 444, 439, 447, and 449 bp in length, respectively, and all exhibited sequencing depth below 10. BLAST analysis revealed that four contigs corresponded to *M. synoviae*, whereas the 449 bp contig was identified as *M. fermentans*. Concurrently, the sequenced data from these samples were reanalyzed using our metabarcoding method pipeline (Qiime2). This method did not detect any reads classifiable to *Mycoplasma*.

## 4 Discussion

PCR is generally considered an effective method for pathogen detection (20). In the Republic of Korea, PCR assays using *Mycoplasma*-specific primer sets are employed alongside bacterial culture to detect *Mycoplasma* contamination in veterinary vaccines. If the PCR result is negative, the vaccine is deemed acceptable (1). If the result is positive, a *Mycoplasma* culture test is conducted. However, PCR has notable limitations, particularly the risk of non-specific amplification under certain conditions. This occurs when primers bind to DNA sequences that are similar—but not identical—to the intended target (6). In the case of combination vaccines containing *E. rhusiopathiae* and viral antigens, the 16S rRNA region of *E. rhusiopathiae* shares sequence similarity with *Mycoplasma*-specific primers, leading to non-specific amplification (Figure 1). This study addresses these limitations by improving sensitivity and accuracy through NGS and bioinformatics-based methods. NGS has emerged as a transformative solution that overcomes the constraints of conventional methods, thereby improving the safety and quality of biopharmaceuticals (21). Regulatory agencies, such as the U.S. Food and Drug Administration and the Ministry of Food and Drug Safety in the Republic of Korea, have adopted NGS-based testing to detect both exogenous and endogenous viruses, thereby expanding detection capabilities and significantly reducing analysis time (22, 23).

In this study, we simulated co-infection scenarios by preparing spiked samples containing *E. rhusiopathiae* and various *Mycoplasma* spp. To distinguish between the two, we used two NGS-based analytical approaches: reference mapping and metabarcoding a widely used approach for bacterial taxonomic analysis (24), typically targeting the V3–V4 hypervariable region of the 16S rRNA gene (25). Since the PCR method used for *Mycoplasma* detection also amplifies the V3–V4 region, we examined whether metabarcoding could be incorporated into the testing process. Empirical results showed that relative abundance values decreased proportionally as *Mycoplasma* concentrations declined in the spiked samples. This pattern illustrates the method’s quantitative characteristics and reproducibility. However, its utility in our specific context was limited. The metabarcoding analysis detected low levels of non-specific sequences, such as those from *Bacillus* and *Staphylococcus*, alongside *Mycoplasma*. Standard 16S rRNA-based metabarcoding typically relies on paired-end sequencing of at least 250 bp to ensure sufficient read overlap for error correction. In this study, however, the V3–V4 region (464 bp) was sequenced using 150 bp paired-end reads, resulting in insufficient overlap between forward and reverse reads. As a result, denoising algorithms such as DADA2 may not have corrected errors completely, potentially leading to the identification of non-specific sequences due to limitations in chimera removal and ASV detection. Future studies should focus on optimizing overlap length to improve metabarcoding accuracy. This could be achieved by targeting a shorter region, such as the V4 alone (250 bp), or by increasing the paired-end read length to 250 or 300 bp. However, such improvements may increase sequencing costs.

In contrast, the two-step reference-mapping method proved to be a superior and highly robust solution. The reference-mapping method used in this study employed a two-step alignment procedure. First, sequencing reads were mapped to the 16S rRNA sequence of *E. rhusiopathiae*, and those mapped reads were removed. In the second step, the remaining reads were aligned to a *Mycoplasma* database, followed by *de novo* assembly using only the reads mapped to *Mycoplasma*. The data demonstrated that both mapping steps were essential for accurate identification. Our data confirmed that both steps are essential for accurate analysis. Omitting the first step makes it difficult to distinguish *E. rhusiopathiae* as the true source of PCR-positive results, whereas omitting the second step results in the generation of an excessive number of contigs and markedly prolonged running times. Therefore, inclusion of both steps is necessary to ensure reliable interpretation (data not shown). Results showed that sequences from *E. rhusiopathiae*, the vaccine’s main component, were successfully removed, enabling more precise downstream analysis. Mapping of samples spiked with *Mycoplasma* revealed that over 90% of total reads corresponding to *A. laidlawii*, *M. fermentans*, *M. orale*, and *M. synoviae* were removed in the first mapping step, while a greater proportion of reads remained for *M. hyorhinis* (Table 4). These results suggest that *Mycoplasma*-positive signals in conventional PCR tests of CSFV-*E. rhusiopathiae* vaccines may stem from non-specific amplification due to sequence similarities between *E. rhusiopathiae* and *Mycoplasma* (26–28).

The results obtained from the negative vaccine samples were inconsistent. While reference-mapping approach produced *mycoplasma*-specific contigs, the metabarcoding method did not detect any reads assignable to *Mycoplasma*. Subsequent culture test yielded no microbial growth, which could support the metabarcoding outcome. However, we cannot exclude the possibility that very small amount of *Mycoplasma* were present at levels insufficient for cultivation, or that laboratory contamination introduced *Mycoplasma*-derived reads. To further investigate this discrepancy, we reanalyzed the sequencing data using shotgun metagenomics tools, Kraken2 and Krona (29–31). In this analysis, reads classified as *Mycoplasma* spp. were observed in the same samples where *Mycoplasma* contigs had been generated by reference-mapping (data not shown).

Between the two NGS-based bioinformatics strategies evaluated, the reference-mapping method was selected as the more appropriate approach. This decision was based not only on the occurrence of non-specific signals in metabarcoding but also on the superior detection of genuine *Mycoplasma*-derived reads in negative vaccine samples, indicating greater analytical sensitivity. Because the method is intended as an alternative to conventional PCR, sensitivity is of paramount importance: in current QC practice, PCR-positive results require culture confirmation, and potential false positives can thus be resolved. By contrast, false negatives pose a critical risk by allowing contaminated products to pass undetected.

Despite these advantages, NGS remains limited by higher costs and the need for specialized bioinformatics expertise. Consequently, molecular methods with comparable sensitivity and specificity but lower operational burden—such as real-time PCR or RPA-CRISPR/Cas12a—may represent more practical long-term solutions for routine testing (32, 33). Nevertheless, in the present context, cross-reactivity was identified as a critical issue specifically in vaccines containing *E. rhusiopathiae* and CSFV. Accordingly, the reference-mapping approach was adopted to address this limitation within an otherwise well-established PCR testing system. Although in this study the NGS-based methods were primarily applied to address the specific issue of cross-reactivity with *E. rhusiopathiae* in conventional PCR, future study will extend beyond this case.

While NGS is an established method for adventitious agent screening in biologics like cell banks (21, 34), its application to mitigate specific PCR cross-reactivity in finished, complex vaccine products represents a significant practical advancement. Unlike raw materials, final vaccine products contain high concentrations of antigens that constitute a challenging analytical matrix. Our two-step mapping strategy is specifically tailored to overcome this matrix effect from *E. rhusiopathiae*, and represents a practical application of NGS to solve a persistent, real-world problem in vaccine lot release testing. This concept may also be extended to national lot release testing of viral vaccines, in which primary removal of major antigens could be followed by adventitious virus detection.

## Data Availability

The original contributions presented in the study are publicly available. This data can be found here: NCBI BioProject database under accession number PRJNA1335287.
